# Characterization of *Escherichia coli* Carrying *mcr*-*1*-Plasmids Recovered From Food Animals From Argentina

**DOI:** 10.3389/fcimb.2019.00041

**Published:** 2019-03-06

**Authors:** Johana E. Dominguez, Diego Faccone, Nathalie Tijet, Sonia Gomez, Alejandra Corso, Mariano E. Fernández-Miyakawa, Roberto G. Melano

**Affiliations:** ^1^Laboratorio de Bacteriología General, Centro de Investigaciones en Ciencias Veterinarias y Agronómicas (CICVyA), Instituto de Patobiología, Instituto Nacional de Tecnología Agropecuaria (INTA), Buenos Aires, Argentina; ^2^Consejo Nacional de Investigaciones Científicas y Técnicas (CONICET), Buenos Aires, Argentina; ^3^Servicio de Antimicrobianos, National and Regional Reference Laboratory in Antimicrobial Resistance, Instituto Nacional de Enfermedades Infecciosas (INEI)-Administración Nacional de Laboratorios e Institutos de Salud (ANLIS) “Dr. C. Malbrán”, Buenos Aires, Argentina; ^4^Public Health Ontario Laboratory, Toronto, ON, Canada; ^5^Department of Laboratory Medicine and Pathobiology, University of Toronto, Toronto, ON, Canada; ^6^Sinai Health System, Mount Sinai Hospital, Toronto, ON, Canada

**Keywords:** *mcr-1* colistin resistance, IncI2 plasmids, *Escherichia coli*, poultry, animals

## Abstract

In this study, we found *mcr-1.1* and *mcr-1.5* genes carried by IncI2 plasmids in a subset of *Escherichia coli* isolates recovered from commercial broiler farms in Argentina. The comparative analysis of the sequences of these plasmids with those described in human clinical isolates suggests that this replicon-type is one of the main *mcr*-disseminator sources in Argentina.

## Introduction

Colistin is a last-resort antimicrobial against multidrug-resistant Gram-negative pathogens. A public health concern about colistin resistance has been risen due to a plasmid-mediated mechanism called *mcr*, described in enterobacteria of clinical and food-animal origin in several countries (Poirel et al., 2017). Fourteen allelic variants of *mcr-1* have been reported lately, designated *mcr-1.1* to *mcr-1.14* (Partridge et al., 2018)*. mcr-1* genes were found in plasmids belonging to different incompatibility groups (IncI2, IncHI2, IncP, IncX4, IncFI, and IncFIB) (Poirel et al., 2017), which mediate their horizontal transfer to different bacterial species.

The global distribution of *mcr* genes in *Escherichia coli* emphasizes the importance of understanding the mechanisms involved in their spread. Rational use of colistin is urgently required to prevent the rapid dissemination of *mcr* to other bacteria and in different niches, including human hospitals and foodborne settings.

In a previous study, we have characterized 149 *mcr-1*-positive *E. coli* isolates recovered from 129 commercial broiler healthy chicken (aged 4–6 weeks) from farms located in several provinces of Argentina (Dominguez et al., 2017). A subset of 10 *E. coli* from that previous study was included in the present work. We describe a comparative analysis of the sequences of their *mcr*-harboring plasmid with those described in human clinical isolates from Argentina.

## Materials and Methods

Ten *mcr-1*-positive *E. coli* isolates were included in this study from healthy chickens recovered from commercial farms located at Entre Rios and Buenos Aires provinces (Table 1). Susceptibility profiles were determined by the agar dilution method with the exception of colistin tested by broth microdilution method. The results were interpreted according to the Clinical and Laboratory Standards Institute guidelines (CLSI, 2017); colistin and tigecycline were interpreted by the 2018 European Committee on Antimicrobial Susceptibility Testing guidelines (http://www.eucast.org). *mcr-1*, ESBL, p*AmpC*, and PMQR-coding-genes were screened by PCR (Anchordoqui et al., 2015; Liu et al., 2016; Albornoz et al., 2017). The genetic relatedness of *E. coli* isolates was studied by PFGE of XbaI-digested genomic fragments. Isolates were also genotyped by multilocus sequence typing (MLST). The allelic numbers and STs were assigned online using http://mlst.warwick.ac.uk/mlst/dbs/Ecoli. Plasmid profile of the isolates was analyzed by S1-PFGE. Sodium azide-resistant *E. coli* J53 was used as a recipient strain in conjugation experiments to study the transferability of the resistance genes. Plasmids were extracted from *mcr-*transconjugants strains using the Qiagen Large-Construct kit (Qiagen) and sequenced using Illumina's MiSeq system. The obtained reads were assembled using CLC Genomics Workbench software (CLCbio, Qiagen), annotated using RAST server (http://rast.nmpdr.org/rast.cgi) and the sequences (gaps were not filled) compared in a pairwise fashion using BRIG (Alikhan et al., 2011). The contigs were also analyzed by ResFinder, PlasmidFinder, and VirulenceFinder tools available from the Center for Genomic Epidemiology website (https://cge.cbs.dtu.dk/services).

**Table 1:** Antimicrobial susceptibility profiles, sequence types (ST) and resistance determinant of *E. coli* isolates.

**Isolates**	Provinces^*^	MIC (μg/ml)^**^													MLST (ST)^***^	***mcr-1* allele**	**ESBL/p*AmpC* genes**	**PMQR genes**	**Plasmids ID**
		**COL**	**AMS**	**CAZ**	**CTX**	**FEP**	**ATM**	**NAL**	**CIP**	**AMK**	**GEN**	**TGC**	**FOS**	**MIN**					
M22607	BA	16 (R)	16 (I)	0.5 (S)	4 (R)	2 (S)	2 (S)	≥64 (R)	32 (R)	2 (S)	0.5 (S)	0.12 (S)	≤0.25 (S)	2 (S)	617	*mcr-1.5*	CTX-M-14	None	pGN2424
M22608	BA	16 (R)	1 (S)	0.12 (S)	0.03 (S)	0.03 (S)	≤0.06 (S)	16 (S)	0.25 (S)	2 (S)	0.5 (S)	0.12 (S)	≤0.25 (S)	4 (S)	1,141	*mcr-1.1*	None	*qnrB*	pGN2415
M22609	BA	8 (R)	64 (R)	4 (S)	64 (R)	8 (I)	32 (R)	≥64 (R)	64 (R)	1 (S)	0.25 (S)	0.25 (S)	64 (S)	4 (S)	410	*mcr-1.1*	CTX-M-2	None	pGN2416
M22610	BA	16 (R)	64 (R)	4 (S)	64 (R)	8 (I)	32 (R)	≥64 (R)	16 (R)	2 (S)	0.5 (S)	0.25 (S)	≤0.25 (S)	32 (R)	155	*mcr-1.5*	CTX-M-2	None	pGN2417
M22611	BA	16 (R)	16 (I)	16 (R)	8 (R)	0.12 (S)	8 (I)	2 (S)	0.008 (S)	2 (S)	0.5 (S)	0.12 (S)	≤0.25 (S)	4 (S)	1,286	*mcr-1.5*	CMY-2	None	pGN2418
M22612	BA	16 (R)	64 (R)	4 (S)	64 (R)	8 (I)	32 (R)	16 (S)	0.5 (S)	2 (S)	4 (S)	0.12 (S)	≤0.25 (S)	0.5 (S)	1,011	*mcr-1.5*	CTX-M-2	*qnrB*	pGN2419
M22613	ER	8 (R)	2 (S)	0.12 (S)	0.03 (S)	0.03 (S)	≤0.06 (S)	16 (S)	0.5 (S)	2 (S)	0.5 (S)	0.12 (S)	0.5 (S)	0.25 (S)	10	*mcr-1.5*	None	*qnrB*	pGN2420
M22614	ER	16 (R)	4 (S)	0.25 (S)	0.12 (S)	0.06 (S)	0.12 (S)	≥64 (R)	0.5 (S)	2 (S)	0.5 (S)	0.25 (S)	0.5 (S)	1 (S)	155	*mcr-1.5*	None	None	pGN2421
M22615	ER	32 (R)	2 (S)	0.12 (S)	0.03 (S)	0.03 (S)	≤0.06 (S)	16 (S)	0.25 (S)	4 (S)	1 (S)	0.12 (S)	0.5 (S)	4 (S)	1,408	*mcr-1.5*	None	*qnrB*	pGN2422
M22616	ER	8 (R)	16 (I)	1 (S)	32 (R)	4 (I)	8 (I)	≥64 (R)	0.5 (S)	2 (S)	16 (R)	0.12 (S)	0.5 (S)	0.5 (S)	Unknown ST	*mcr-1.5*	CTX-M-14	None	pGN2423

* *BA, Buenos Aires; ER, Entre Ríos*.

** *COL, colistin; AMS, ampicillin-sulbactam; CAZ, ceftazidime; CTX, cefotaxime; FEP, cefepime; ATM, aztreonam; NAL, nalidixic acid; CIP, ciprofloxacin; AMK, amikacin; GEN, gentamicin; TGC, tigecycline; FOS, fosfomycin; MIN, minocycline. R, resistant; I, intermediate, S, susceptible by the agar dilution method with the exception of colistin tested by broth microdilution method*.

*** *MLST, Multilocus Sequence Typing*.

## Results and Discussion

All *E. coli* isolates were positive for *mcr-1* and exhibited resistance to colistin. Some isolates also exhibited a multidrug-resistant (MDR) phenotype including resistance to expanded-spectrum cephalosporins, quinolones, gentamicin, and minocycline, but all of them were susceptible to amikacin, tigecycline, and fosfomycin. *mcr*-1-positive isolates were determined to carry ESBL (5/10, *bla*_CTX−M−2_ or *bla*_CTX−M−14_), p*AmpC* (1/10, *bla*_CMY−2_), and PMQR (4/10, *qnrB*) genes (Table 1).

All isolates exhibited different PFGE profiles, indicating that they were genetically unrelated (Supplementary Figure 1). Supporting the PFGE results, most of the isolates had different STs. Only two of them, recovered in both provinces (Entre Rios and Buenos Aires), were ST155. Four isolates belonged to clonal complex 10 (CC10): one ST10, two single locus variants (ST1141 and ST1286), and one double locus variants (ST617) of ST10 (Table 1). *E. coli* CC10 isolates are globally recovered from food-producing animals and human samples, however, it is particularly frequent in livestock animals as susceptible or multidrug-resistant isolates (ESBL and/or p*AmpC* producers) (El Garch et al., 2017). One isolate was ST410, this ST was previously found in a *mcr-1*-positive clinical *E. coli* isolate in Argentina (Tijet et al., 2017), which was defined as a hyperepidemic clone and the possible founder of the disseminated CC23 (Turrientes et al., 2014).

A diverse plasmid content was found in the isolates by S1-PFGE. All isolates harbored a ca. 61-kb plasmid, present also in all the *mcr-1*-transconjugant strains conferring them only resistance to colistin (Supplementary Figure 2).

**Figure 1:**
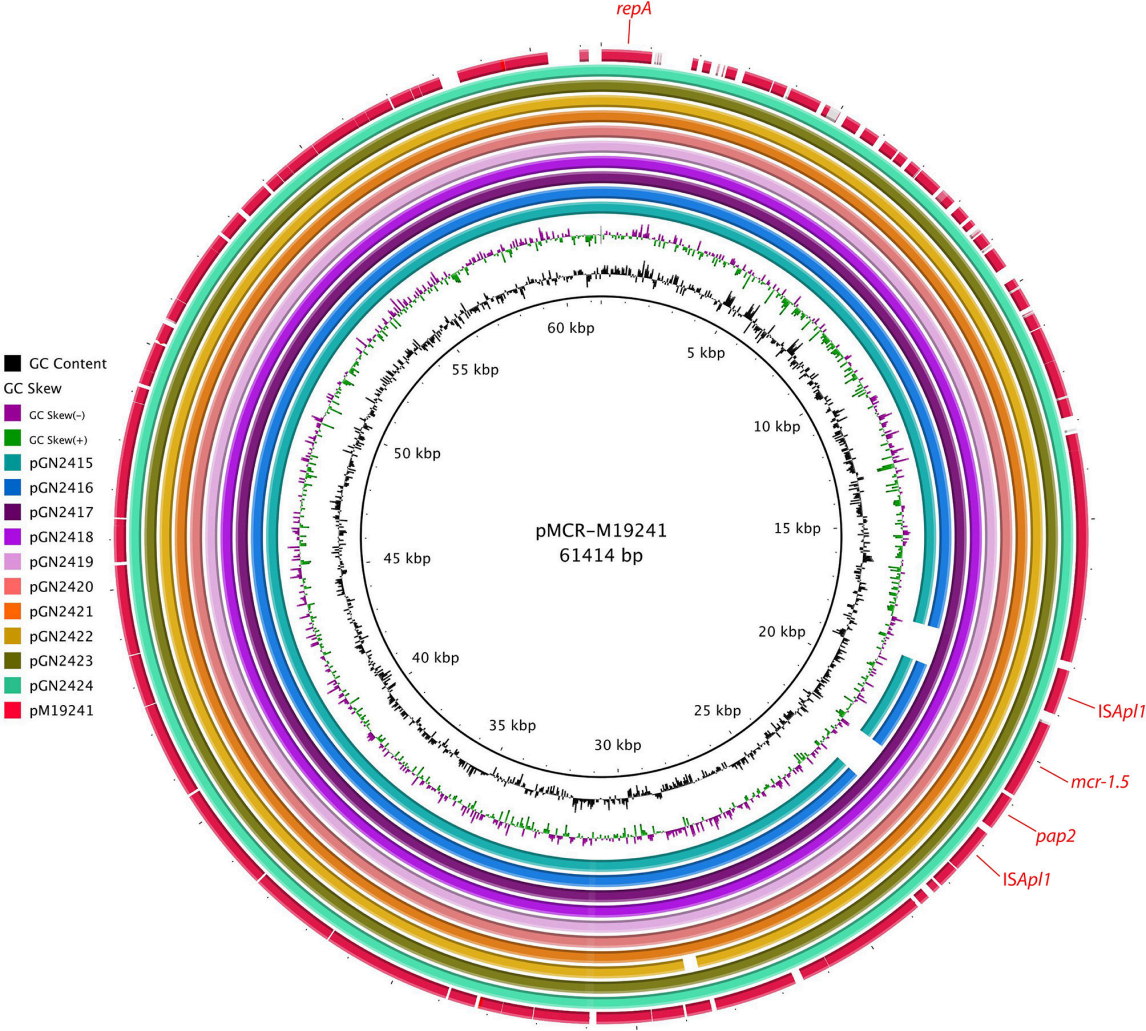
BRIG alignment of *mcr-1*-positive plasmids recovered from *E. coli* isolates. The location of *mcr-1.5* and the *repA* plasmid replication gene are indicated. The comparisons are made relative to plasmid pMCR-M19241 (GenBank KY471311), recovered from a clinical *E. coli* isolate from Argentina.

Assembling of short reads yields between 6 and 8 contigs from 8 isolates, while 2 isolates rendered 4 contigs. In all cases the calculated total length, ca. 61 kb, was in agreement with the plasmid sizes estimated by S1-PFGE. Eight of the ten plasmids analyzed had the same variant described as *mcr-1.5*, found in clinical isolates of Argentina (Tijet et al., 2017), and the remaining two contained the *mcr-1.1* variant. Blast-based query revealed that all plasmids belonged to the IncI2 incompatibility group and none of them carried additional resistance or virulence genes. A comparison of the plasmids with pMCR-M19241 (obtained from human clinical isolate, GenBank KY471311) (Tijet et al., 2017) shows that eight of them (pGN2417 to pGN2424) contained two copies of the insertion sequence IS*ApI1* flanking the *mcr-1.5/pap2* fragment, which might facilitate the transfer of *mcr-1* between DNA molecules. IS*ApI1* was not present in pGN2415 and pGN2416 (Figure 1). Plasmids had a typical backbone responsible for its replication, maintenance, and transfer (Sun et al., 2016). Main differences observed between the plasmids were mainly due to reorganization of the *pilV* shufflon (data not shown).

*mcr-1*-encoding IncI2 plasmids have previously been reported in studies from Asia (mainly in China and Japan), Europe and the U.S (Ohsaki et al., 2017). Moreover, different plasmid types harboring *mcr-1* have been reported in South-America, in isolates recovered from food animals, clinical samples and environmental reservoirs (Delgado-Blas et al., 2016; Fernandes et al., 2017; Monte et al., 2017; Rossi et al., 2017; Saavedra et al., 2017). Our results, together with previous studies (Tijet et al., 2017), suggest that in Argentina the spread of *mcr-1* in *E. coli* isolates from animals and humans could be mainly mediated by IncI2-type plasmids.

## Conclusion

These findings point toward effective dissemination of the *mcr-1* gene in Argentina by efficient horizontal transfer almost exclusively by IncI2 type plasmids (Tijet et al., 2017). Our recent study suggested that a group of *mcr-1*-positive plasmids with same backbones are present in poultry farm *E. coli* isolates as well as human clinical *E. coli* isolates. Here, we report comparative genomics of over 10 representative *mcr-1*-bearing plasmids. These findings expand the scenery of *mcr-1*-harboring plasmids in Argentina.

## Author Contributions

JD, DF, SG, AC, MF-M, and RM participated in the design of the study. JD, DF, and NT performed the experiments. JD, DF, SG, AC, and RM analyzed the data. JD and MF-M collected *E. coli* strains. JD, DF, NT, SG, AC, MF-M, and RM wrote the paper. All authors contributed to the critical revision of the manuscript and have seen and approved the final draft. All authors read and approved the final manuscript.

### Conflict of Interest Statement

The authors declare that the research was conducted in the absence of any commercial or financial relationships that could be construed as a potential conflict of interest.

## Supplementary Material

Supplementary Figure 1 The genetic relatedness of *mcr-1-*positive *E. coli* isolates. PFGE: Gel order; Line 2, M22607; 3, M22608; 4, M22609; 5, M22610; 6, M22611; 7, M22612; 8, M22613; 9, M22614; 10, M22615; 11, M22616; Lanes 1 and 12, *S*. Branderup.

Supplementary Figure 2 Plasmid profile. S1-PFGE: Gel order; Line 2, M22608; 3, M22608-TC; 4, M22609; 5, M22609-TC; 6, M22610; 7, M22610-TC; 8, M22611; 9, M22611-TC; 10, M22612; 11, M22612-TC; 12, M22613; 13, M22613-TC; 14, M22615; 15, M22615-TC; 16, M22616; 17, M22616-TC; 19, M22607; 20, M22607-TC; 21, M22614; 22, M22624-TC; Lanes 1, 18, and 23, *S*. Branderup. TC: transconjugants obtained using *E. coli* J53 AZ^R^ as the recipient strain. Red arrows highlight plasmids containing the *mcr-1* gene.
