# Supplementary figures and images for: Characterization of Escherichia coli Carrying mcr-1-Plasmids Recovered From Food Animals From Argentina
Source: Front Cell Infect Microbiol. 2019 Mar 6;9:41. doi: 10.3389/fcimb.2019.00041 (PMC6414435; doi:10.3389/fcimb.2019.00041)

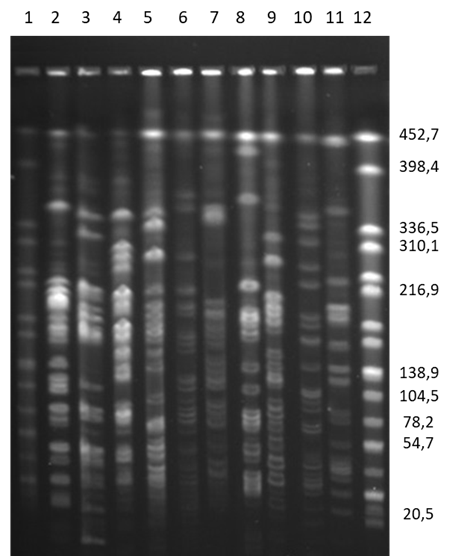

Supplement: Supplementary Figure 1 — The genetic relatedness of mcr-1-positive E. coli isolates. PFGE: Gel order; Line 2, M22607; 3, M22608; 4, M22609; 5, M22610; 6, M22611; 7, M22612; 8, M22613; 9, M22614; 10, M22615; 11, M22616; Lanes 1 and 12, S. Branderup. [file Image_1.TIF]

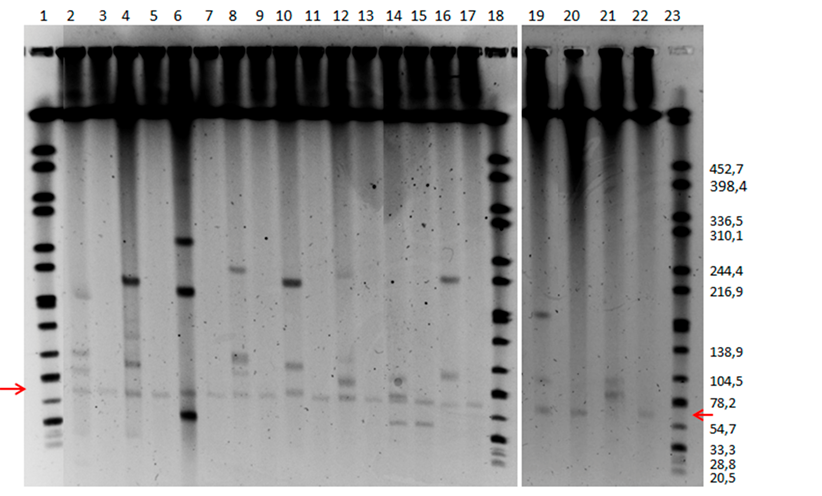

Supplement: Supplementary Figure 2 — Plasmid profile. S1-PFGE: Gel order; Line 2, M22608; 3, M22608-TC; 4, M22609; 5, M22609-TC; 6, M22610; 7, M22610-TC; 8, M22611; 9, M22611-TC; 10, M22612; 11, M22612-TC; 12, M22613; 13, M22613-TC; 14, M22615; 15, M22615-TC; 16, M22616; 17, M22616-TC; 19, M22607; 20, M22607-TC; 21, M22614; 22, M22624-TC; Lanes 1, 18, and 23, S. Branderup. TC: transconjugants obtained using E. coli J53 AZR as the recipient strain. Red arrows highlight plasmids containing the mcr-1 gene. [file Image_2.TIF]
